# Colonoscopic surveillance in Lynch syndrome: guidelines in perspective

**DOI:** 10.1007/s10689-024-00414-y

**Published:** 2024-07-27

**Authors:** Joaquín Castillo-Iturra, Ariadna Sánchez, Francesc Balaguer

**Affiliations:** 1 Department of Gastroenterology, Hospital Clínic de Barcelona, Centro de Investigación Biomédica en Red de Enfermedades Hepáticas y Digestivas (CIBEREHD), Institut d’Investigacions Biomèdiques August Pi i Sunyer (IDIBAPS), Barcelona, Spain; 2 Facultat de Medicina i Ciències de la Salud, Universitat de Barcelona (UB), Barcelona, Spain

**Keywords:** Lynch, Colonoscopy, Surveillance, Colorectal cancer

## Abstract

Lynch syndrome predisposes to a high risk of colorectal cancer and colonoscopy remains the primary preventive strategy. The prevention of colorectal cancer through colonoscopy relies on identifying and removing adenomas, the main precursor lesion. Nevertheless, colonoscopy is not an optimal strategy since post-colonoscopy colorectal cancer remains an important issue. In continuation of a 2021 journal review, the present article seeks to offer an updated perspective by examining relevant articles from the past 3 years. We place recent findings in the context of existing guidelines, with a specific focus on colonoscopy surveillance. Key aspects explored include colonoscopy quality standards, timing of initiation, and surveillance intervals. Our review provides a comprehensive analysis of adenoma-related insights in Lynch syndrome, delving into emerging technologies like virtual chromoendoscopy and artificial intelligence-assisted endoscopy. This review aims to contribute valuable insights into the topic of colonoscopy surveillance in Lynch syndrome.

## Introduction

The identification and removal of adenomas, acknowledged as the main colorectal cancer (CRC) precursor lesions, are thought to play a crucial role in CRC prevention in Lynch syndrome (LS). However, both prospective and retrospective studies highlight a significant number of LS carriers developing CRC despite regular colonoscopy surveillance. The Prospective Lynch Syndrome Database (PLSD) study described cumulative incidences at 70 years by gene of 46%, 35%, 20% and 10% for *MLH1*, *MSH2*, *MSH6* and *PMS2* pathogenic variant carriers, respectively [1]. This phenomenon is attributed to factors such as missed or incomplete resected lesions during colonoscopy, rapid progression of lesions that occur after a normal colonoscopy, and the potential existence of an adenoma-free pathway to CRC that omits the adenoma phase [2, 3]. This hypothesis relies on mechanistic differences in adenoma formation in *MLH1* carcinogenesis through inactivation of *CTNNB1*, and a lower incidence of adenomas in these carriers. However, the extent to which colonoscopy effectively prevents CRC in LS remains an ongoing controversy. In 2021, a progress report on LS CRC endoscopic surveillance was published in this journal [4]. During the last three years a significant amount of new data have emerged assessing the role of surveillance colonoscopy in LS. In this report we discuss the main new articles in this field, focusing on the new knowledge that can potentially change our clinical practice and decision-making.

## Role of colonoscopy in CRC prevention in Lynch syndrome

Colonoscopy prevents CRC mainly by the detection and removal of precursor lesions in the colorectum, known as polyps. Adenomas are the main precursor of CRC in LS, and evidence suggests that serrated lesions do not play a significant role [5]. However, in the last decade a large amount of evidence has shown that the effectiveness of colonoscopy in CRC prevention is heterogenous due to variable quality between procedures. As an example, in the setting of population-based screening colonoscopy, the adenoma miss rate, defined as the proportion of missed adenomas detected upon a second examination of the colon can be as high as 25% for small adenomas (< 10 mm) [6, 7].

Several factors contribute to colonoscopy quality, including bowel preparation, withdrawal time, cecal intubation, appropriate polypectomy technique, endoscopist adenoma detection rate (ADR), among others. Notably, ADR stands out as the most extensively studied colonoscopy quality indicator. ADR is defined as the proportion of screening colonoscopies performed by a physician that detects at least one histologically confirmed adenoma. It is inversely associated with the risk of post-colonoscopy CRC (PCCRC) and CRC-related mortality [8], establishing itself as the best key quality indicator described so far. Consequently, ADR serves as a benchmark for evaluating endoscopist performance, distinguishing between lower and higher performers with direct clinical implications. Minimum ADR targets in average-risk individuals are 25% for men and 15% for women in colonoscopy-based programs, and 45% in fecal immunochemical test-based ones [9]. More importantly, an improvement in endoscopists’ ADR has demonstrated a correlation with a reduction in CRC incidence and mortality [10].

### Adenoma features in Lynch syndrome

Adenoma’s features in LS have shown to be distinct from the average-risk population. They are often non-polypoid or flat in shape and more frequently located in the proximal colon, making their detection challenging for endoscopists [11, 12]. Furthermore, adenomas in LS tend to exhibit high-grade dysplasia more frequently [11] even in small (< 5 mm) lesions, indicating a rapid progression of these precursor lesions into cancer. Six studies with back-to-back colonoscopies (where two consecutive same-day colonoscopies are performed) have revealed a notable miss rate of adenomas in LS [13–18]. Notably, an adenoma miss rate over 48% was observed in all six studies, revealing a remarkably high rate compared to the adenoma miss rate described in the average-risk population. Importantly, none of these studies compared the adenoma miss rate between different genes. These observations suggest that missed adenomas in LS surveillance colonoscopies are frequent events and considering their advanced features even when they are small, this is thought to be a plausible cause of the inefficacy of colonoscopy preventing CRC.

### Yield of surveillance colonoscopy in Lynch syndrome

Prevalence and incidence of adenomas in LS remains poorly understood. Both the fact that the effectiveness of colonoscopy for the detection of adenomas is variable, and the different definitions of the adenoma detection rate in published studies, makes very difficult the estimation of the true prevalence and incidence of adenomas. ADR, established and validated for screening colonoscopy in average-risk population defines the performance of an endoscopist. However, a similar figure in LS has not been established. The fact that the age span of LS surveillance is very wide (starting at age 24–35 years-old), and the fact that LS carriers undergo colonoscopies every 1–3 years, lead to a difficult scenario to define a benchmark as clear as ADR. The concept of ADR in LS research articles describes adenoma detection in a single colonoscopy or in multiple colonoscopies, rather than evaluating the detection rate of the endoscopist. Consequently, ADR, as the most important quality indicator in screening colonoscopy, is not directly comparable to ADR in LS surveillance. Another potential limitation of the reviewed studies is the lack of control for the impact of acetylsalicylic acid as a chemopreventive agent, which could potentially modify ADR in LS.

The most recent evidence regarding the adenoma detection in LS is summarized in Table 1, where adenoma detection at first colonoscopy, during surveillance colonoscopies and overall adenoma detection (individuals diagnosed with at least one adenoma) are detailed. In 2020, Engel et al. conducted a multicenter cohort study including 2,747 individuals with LS, 1,038 (37.8%) with a previous CRC. At index colonoscopy, the frequency of prevalent adenomas in that study was only 10.2% [19]. More recently, five studies have described the adenoma detection rates with surprisingly variable results. Sánchez et al., in a multicenter cohort of 893 LS individuals without previous CRC in Spain and The Netherlands reported 19.8% of adenoma detection at index colonoscopy [20]. Del Carmen et al., in a single-centered study with 163 individuals, found a 21.4% of adenomas at index colonoscopy [21] and Aronson et al., in a cohort of 429 individuals, reported a 19.8% of adenoma detection at first colonoscopy and 17.5% in subsequent colonoscopies [22]. Alric et al. described the adenoma characteristics of young LS carriers, including 708 individuals under the age of 50 years, with an adenoma detection of 19.2% at first colonoscopy, 20.5% in subsequent colonoscopies and an overall adenoma detection of 46.6% [23]. Finally, Miyakura et al., recently described figures of 25% adenoma detection rate at index colonoscopy [24]. In summary, these studies show that adenoma detection at index colonoscopy varies greatly from 10.2 to 27%. Although these are studies with different populations and background, the difference in adenoma detection is striking and probably reflects the variability of colonoscopy as a detection method, which will be discussed in the following section of this review.

**Table 1:** Adenoma detection in Lynch syndrome

	*N*	Population description	Adenoma detection at index colonoscopy	Adenoma detection at subsequent colonoscopy	Overall adenoma detection	Adenoma incidence
Vale Rodrigues et al. (2018) [32]	147	High-risk cancer clinic, Lisboa, Portugal. No previous CRC. 2005–2016	-	-	54%	55.2%
Goverde et al. (2020) [26]	264	Single centered, Rotterdam, the Netherlands. 1985–2017	27% (*MLH1* 27%, *MSH2* 23%, *MSH6* 26%, *PMS2* 36%)	-	46%	-
Engel et al. (2020) [19]	2747	Germany, the Netherlands, and Finland. 1984–2015	10.2%	-	-	10-year cumulative incidence: *MLH1* 32.2% (95% CI, 29.2–35.2%) *MSH2* 44.2% (95% CI, 40-48.4%) *MSH6* 38.4% (95% CI, 30.8–45.9%)
Bucksch et al. (2022) [27]	864	German Consortium for Familial Intestinal Cancer.	-	-	-	Cumulative adenoma risk at 50 years: 84.1% (95% CI, 74.7–91.5%)
Sánchez et al. (2022) [20]	893	High-risk cancer clinics, Spain and the Netherlands. No previous CRC. 1987–2019	19.8%	16.5%	45.6%	10-year cumulative incidence: 60.6% (95% CI, 55.5–65.2%)
Del Carmen et al. (2023) [21]	163	Single centered, MD Anderson, United States. 1997–2020	21.4%	-	-	-
Aronson et al. (2023) [22]	429	Decentralized hospitals, Toronto, Canada.	19.8%	17.5%	46.6%	-
Sleiman et al. (2023) [25]	132	Cleveland Clinic, Ohio, United States. No previous CRC. 1972–2020			33.9% (before LS diagnosis) − 40.9% (after LS diagnosis)	
Alric et al. (2023) [23]	708	High-risk cancer clinics, Paris, France. LS carriers < 50 years old. 2010–2019	19.2%	20.5%	36.3%	-
Miyakura et al. (2023) [24]	309	Multicenter, Japan.	25%	-	57%	

Gene-specific differences in adenoma detection in LS are still controversial. Unlike what was published by Engel et al. in the so-called three countries study (Finland, Germany and Netherlands), which described a lower incidence of adenomas and advanced adenomas in *MLH1* carriers (*MLH1* vs. *MSH2*; 32.2% and 7.7% vs. 44.2% and 17.9%, respectively), many other recent studies have failed to clearly show such difference [21, 25–27]. This is a key aspect to clarify since if there are differences in the incidence of adenomas between genes, it could be important in the surveillance strategy.

Finally, over the past few years, there has been a growing interest in the use of immune checkpoint blockade (ICB) therapy for mismatch repair-deficient tumors [28–30]. Metastatic and localized mismatch repair-deficient tumors are exquisitely sensitive to ICB. However, it remains unclear whether ICB can prevent the development of pre-malignant neoplasia in patients with LS. Harrold et al. recently analyzed the impact of ICB treatment on pre-malignant polyp (serrated adenomas were included in this definition) detection and the development of metachronous cancer in 61 cancer patients with LS. Contrary to expectations, pre-malignant polyps were identified in 39% of patients post-ICB treatment, surpassing the baseline detection rate, and 12% developed a new cancer. Interestingly, the authors observed higher rates of cutaneous metachronous tumors and lower rates of visceral tumors after ICB, suggesting that visceral neoplasia may be decreased by ICB exposure. Notably, 91% of new cancers and a subset of premalignant neoplasia were mismatch repair deficient [31]. These data suggest that ICB treatment of LS-associated tumors does not eliminate risk of new colorectal neoplasia development, and LS-specific surveillance strategies should be continued.

### Colonoscopy quality indicators in Lynch syndrome

Missed lesions are considered as a primary cause of PCCRC in the general population. When combined with procedural factors such as incomplete examinations, poor bowel preparation, incomplete polyp removal, and deviations from recommended surveillance intervals, they can account for a substantial majority of PCCRC cases [33]. Although limited evidence exists regarding quality indicators in LS, the studies suggest that colonoscopy quality in LS surveillance is heterogeneous. Stoffel et al. investigated the adherence to recommended surveillance intervals in 181 LS carriers, revealing that 27% had intervals longer than the 2 years interval recommendations [34]. In a study comparing bowel cleansing preparation in LS colonoscopies, van Vugt van Pinxteren et al. observed suboptimal colon cleansing regardless of the preparation used in 72.4% of cases [35]. In these studies, bowel prep is usually graded as excellent, good, fair and poor, not using the Boston Bowel Preparation Score, which is the most used in clinical practice. Haanstra et al. examined the quality of the previous colonoscopies from 31 cases of PCCRC, reporting incomplete examinations in 16% of cases and lack of information in completion in 13%. Adequacy of preparation was not reported in 54% of cases, and incomplete removal of adenomas was suspected in 67% of cases where an adenoma was found in the same part of the colon during previous colonoscopy [36].

Recently, Sánchez et al. conducted a multicenter study involving the data of 4,177 colonoscopies allowing a comprehensive evaluation of quality indicators showing that only 50.7% of carriers had all their examinations performed within 2 years, and overall, only 28.2% of carriers undergoing complete colonoscopies with adequate bowel preparation and intervals of less than 2 years (of note, in Spain the recommendation for LS surveillance was 2 years irrespective of the affected gene). In this study adequate bowel preparation, cecal intubation and the use of chromoendoscopy were associated with a significantly improvement on adenoma detection. In order to evaluate the impact of colonoscopy quality indicators on PCCRC prevention, the authors performed an emulated target trial comparing the incidence of CRC between the first and second surveillance colonoscopy. PCCRC risk was significantly lower when colonoscopies were performed in an interval shorter than 3-year [odds ratio (OR), 0.35; 95% confidence interval (CI), 0.14–0.97]. For the other quality indicators, the authors observed a non–statistically significant reduction in PCCRC risk: previous complete examination (OR, 0.16; 95% CI, 0.03–1.28), previously adequate bowel preparation (OR, 0.64; 95% CI, 0.17–3.24), and previous use of a high-definition scope (OR, 0.37; 95% CI, 0.02–2.33). One notable limitation in this study was the absence of endoscopist’s ADR as a quality indicator [20]. Miyakura et al., in a retrospective multicenter study involving 309 LS carriers, investigated the influence of endoscopists’ experience on the detection of colorectal lesions. The study observed that highly experienced endoscopists with more than 5,000 colonoscopies detected a higher mean number of adenomas and intramucosal cancer in LS individuals [24].

Finally, Møller et al. analyzed and compared the CRC incidence in two large cohorts of individuals with LS included in the PLSD (*n* = 8,153) and the International Mismatch Repair Consortium (IMRC, which contained data from 5,255 families with LS). In this study, the authors assumed that all the PLSD cohort underwent colonoscopy surveillance with polypectomy and that the IMRC did not. Surprisingly, the cumulative incidences of CRC were higher in the PLSD vs. IMRC carriers, suggesting that colonoscopy surveillance does not impact CRC incidence at all [37]. The results of this study should be taken with caution, since data on the actual performance of colonoscopy was not available, and considering the evidence discussed above, this is a potential bias of the study that was not taken into consideration.

In summary, quality standards are often not met in LS surveillance with likely direct consequences for adenoma detection and PCCRC incidence. Based on the described studies, guidelines from the European Society of Gastrointestinal Endoscopy (ESGE) [38] and National Comprehensive Cancer Network (NCCN) recommend that colonoscopy surveillance should be performed in dedicated centers and likely, by more experienced endoscopists. However, more evidence is needed to establish the impact of quality colonoscopy indicators and the PCCRC risk in LS and define specific quality indicators in this population.

### Virtual chromoendoscopy

Chromoendoscopy (CE) involves the application of dye over the mucosa (dye-based) or the use of light filters (virtual chromoendoscopy) to enhance the visualization of mucosal lesions. In the average-risk population, both dye-based and virtual CE have demonstrated increased adenoma detection. However, in LS, evidence supporting CE is less robust, hampered by methodological issues. Many studies supporting CE rely on non-randomized back-to-back designs, introducing observational bias due to the inherent nature of the approach (where the second inspection of the colon renders by itself a high miss adenoma rate). Although two large multicenter randomized parallel trials failed to show statistically significant benefits for dye-based CE compared to white-light endoscopy (WLE), a noticeable trend towards increased detection of flat adenomas was observed (Table 2) [39, 40]. Moreover, a meta-analysis on dye-based CE in LS showed no apparent increase in ADR compared to WLE. However, the evidence was assessed as low-quality due to wide confidence intervals and observed differences in study methodologies [41]. Current guidelines suggest potential benefits of CE but emphasize the need to weigh its use against consideration of costs, training and practicality [38].

**Table 2:** Detection rate of adenomas under chromoendoscopy in randomized trials

	*N*	CE type	WLE adenoma detection	CE adenoma detection
Bisschops et al. (2017) [43]	61	HD-WLE vs. I-SCAN	32% (HD-WLE→ I-SCAN)	30% (I-SCAN → HD-WLE)
Haanstra et al. (2019) [39]	246	WLE vs. Dye-based CE	27%	30%
Rivero-Sánchez et al. (2020) [40]	256	HD-WLE vs. Dye-based CE	28.1%	34.4%
Houwen et al. (2022) [42]	332	HD-WLE vs. LCI	25.6%	36.3%

CE: chromoendoscopy; WLE: white light endoscopy; HD: high definition; LCI: Linked Colour Imaging

In contrast to the time-consuming dye CE, virtual CE appears to be a compelling approach whose role has not yet been well established. A recent multicenter randomized controlled trial by Houwen et al. compared Linked Colour imaging (LCI), an image-enhancing technique, with white-light high-definition endoscopy (HD-WLE) in 332 LS individuals. The LCI group exhibited a significantly higher detection of proximal adenomas (28.1% vs. 18.6%; *p* = 0.04) and ≤ 5 mm adenomas (32.5% vs. 22.1%; *p* = 0.03), contributing to an overall higher detection rate of adenomas (36.3% vs. 25.6%; *p* = 0.04). No significant differences were found in the detection of flat adenomas (16.9% vs. 11.6%, *p* = 0.18), adenomas *≥* 5 mm and serrated lesions. It is noteworthy that this study observed a very high adenoma detection rate, which the author attributes to the exclusive participation of experienced endoscopists and a potential observational bias [42]. Consequently, stronger recommendations for the use of LCI are anticipated in future guidelines. However, additional studies are essential to explore whether other forms of virtual CE, besides LCI, offer benefits in this setting.

### Role of artificial intelligence

Endoscopic image analysis using artificial intelligence (AI) has made its way into colonoscopy through computer-aided polyp detection (CADe) and diagnosis (CADx). Currently, several AI systems are already integrated into endoscopy units. The current method in AI-assisted detection involves real-time detection by flagging suspicious lesions. This technology has demonstrated a significant improvement in the ADR in the average-risk population, particularly by increasing the detection of small, non-polypoid, and distal adenomas [44]. The latest meta-analysis on this subject, reviewing 21 randomized trials, found that the CADe group exhibited an increased ADR compared to standard colonoscopy (44% vs. 35.9%; RR = 1.24; 95% CI = 1.16 to 1.33) with a number needed to scope of 13.5 to detect 1 additional adenoma [45].

The first report of the use of this tool in LS surveillance comes from a pilot study published by Hüneburg et al. In this study, a randomized controlled trial was performed aimed at investigating the diagnostic performance of AI-assisted colonoscopy (using CAD-eye, Fujifilm) in comparison to HD-WLE, including 96 individuals with LS. In this trial a non-significant increase in ADR was found for the AI group (31.3% vs. 26.1%; *p* = 0.379), as well for advanced adenoma detection rate (8% vs. 4.3%; *p* = 0.063). In addition, AI-assisted colonoscopy detected a higher proportion of flat adenomas (56.6% vs. 20%; *p* = 0.018), and a higher per-colonoscopy proportion of flat adenomas (8% vs. 4.3%; *p* = 0.063) [46]. Data from a randomized controlled trial have been recently published by Ortiz et al. [47] This trial, involving 414 LS carriers, aimed at comparing the performance of CADe (GI Genius™, Medtronic) vs. HD-WLE. No significant differences were found neither in the mean number of adenomas per colonoscopy (0.64 vs. 0.64; *p* = 0.42), nor the ADR (33.3% vs. 37.1%; *p* = 0.47).

More data are needed regarding this matter, however considering these results and the data from colonoscopy outside the LS setting, AI-assisted colonoscopy seems a promising approach for LS surveillance that needs further investigation and development.

## Colonoscopy surveillance strategy in LS

LS is currently understood as a four clinically distinct syndromes with consistent genotype-phenotype associations. Since CRC lifetime risk varies depending on the mismatch repair gene involved, screening guidelines are evolving to become gene specific. Age of initiation and colonoscopy surveillance intervals are critical factors in the effect of colonoscopy on the prevention and early diagnosis of CRC. However, current recommendations by professional societies vary regarding these two aspects (Table 3). The variances among these guidelines highlight the challenge of deriving screening interval recommendations directly from existing data in the absence of prospective studies. The largest study describing CRC risk according to different intervals between colonoscopies (the so-called “three countries study”) did not observe significant differences [48]. European guidelines tend to recommend a later initiation of the surveillance and longer intervals compared to North American guidelines. However, both seek to provide personalized recommendations for *MLH1*, *MSH2*, *MSH6* and *PMS2* carriers. The most recent evidence analyzing age and time interval is summarized in this section.

**Table 3:** Recent guidelines for colonoscopy surveillance in Lynch syndrome

	Gene	Interval	Age of initiation
European Society of Gastrointestinal Endoscopy 2019 [38]	*MLH1/MSH2*	2 years	25 years
	*MSH6/PMS2*	2 years	35 years
British Society Gastroenterology, Association of Coloproctology of Great Britain and Ireland and United Kingdom Cancer Genetics Group 2019 [49]	*MLH1/MSH2*	2 years	25 years
	*MSH6/PMS2*	2 years	35 years
European Hereditary Tumour Group and European Society of Coloproctology 2020 [50]	*MLH1/MSH2*	2–3 years^a^	25 years
	*MSH6*	2–3 years^a^	35 years
	*PMS2*	5 years	35 years
National Comprehensive Cancer Network 2023^d^	*MLH1/MSH2*	1–2 years	20–25 years^b^
	*MSH6/PMS2*	1–3 years	30–35 years^c^

a. 2 years is recommended in patients with previous CRC

b. Or 2–5 years prior to the earliest CRC if it is diagnosed before the age of 25 years

c. Or 2–5 years prior to the earliest CRC if it is diagnosed before the age of 30 years

d. www.nccn.org

Recently, Aronson et al. conducted a study to assess the relationship between colonoscopy screening intervals and CRC risk in 429 LS carriers from the Familial Gastrointestinal Cancer Registry (Zane Cohen Centre, Sinai Health System, Toronto, Canada). The authors employed an instrumental variable approach to evaluate the association between screening intervals and CRC. First, they modeled the association of screening intervals between two consecutive colonoscopies (categorized as 1 year, 1–2 years, 2–3 years, or > 3 years) with the number of detected adenomas, predicting the total number of adenomas detected in each screening interval for all patients. Second, they evaluated the association between the predicted number of adenomas with the time to CRC. The study revealed an association between shorter colonoscopy intervals and a higher number of adenomas detected. There was a clear trend towards a reduction in CRC incidence, especially when comparing 1–2 years to more than 3 years intervals. A decrease in 10-year CRC risk of 11.3% for any new adenoma detected (HR = 0.88, CI = 0.78–0.99) was observed [22]. However, as noted in an editorial by Biller and Ng, this observation may be particularly relevant for *MLH1/MSH2* carriers, constituting more than 70% of the cohort [51]. These findings support a 1–2-year screening interval for a higher adenoma detection and CRC risk reduction. Another notable observation was that individuals with no history of adenomas, comprising 53.4% of the cohort, exhibited a higher CRC incidence compared to those with a history of adenomas. The authors hypothesize that this fact may be the so-called adenoma-free CRC pathway rather than missed adenomas from previous colonoscopy. However, the study is limited by a lack of comprehensive investigation into colonoscopy quality indicators, which were assessed only by the year in which the colonoscopy was performed, academic vs. non-academic hospitals and the number of polyps not submitted to pathology, which are not well-recognized colonoscopy quality indicators. Therefore, given the variability in the detection of adenomas mentioned previously, we cannot be sure that the population without adenomas in this study was a population with adenomas that were missed.

Limited studies have evaluated *MSH6* and *PMS2* colonoscopy surveillance, and recommendations are based on indirect data. In this context, Andresdottir et al. conducted a study in the Icelandic population where massive genotyping and genealogic information has allowed and accurate assessment of the prevalence of LS [52]. As previously published, three founder mutations, one in *MSH6* and two in *PMS2* accounts for the majority of LS in Iceland. The purpose of this study was to describe the metachronous CRC incidence by including all individuals with variants in *MLH1*, *MSH2*, *MSH6*, and *PMS2* and cross-referencing with CRC diagnoses and colon polyp diagnoses from the Icelandic Cancer Registry from 1955 to 2017. Of note, this study represents the largest study performed on metachronous CRC risk in unscreened *MSH6* and *PMS2* carriers. Interestingly, none of the *MSH6* or *PMS2* carriers had a metachronous CRC, with only one CRC diagnosed in an *MSH2* carrier. Significantly more *MSH6* carriers had a history of advanced adenomas as compared with *PMS2* carriers (44% vs. 12%, *p* = 0.005). The median age at CRC diagnosis was 60 years (IQR 55–72; range 41–90) for *MSH6* carriers and 64 years (IQR 54–73, range 40–94) for *PMS2* carriers [53]. In line with these findings, Sleiman et al. recently described the prevalence and incidence of neoplasia in a cohort of 132 LS carriers during surveillance colonoscopy at the Cleveland Clinic. Interestingly, all incident CRC (*n* = 3) occurred in *MSH2* and *MLH1* carriers and only 1 (0.7%) while under surveillance [25]. Overall, these findings emphasize that *MSH6/PMS2* carriers have a low risk of metachronous CRC and develop CRC at a later age, reinforcing a delayed initiation of surveillance might be adequate.

In contrast, certain cohorts, such as the study conducted at the Memorial Sloan Kettering Cancer Center involving 243 *MSH6* and *PMS2* carriers, emphasize a younger age at diagnosis of CRC, with a median age of 51.5 years (range 27–80). Notably, 16% of LS carriers in this cohort were diagnosed before the age of 35 [54]. While the early onset of CRC in these cases may be influenced by various factors, more conservative recommendations, like the latest NCCN guidelines, have responded by lowering the recommended age to initiate colonoscopy to 30–35 years. Additionally, these guidelines suggest considering the initiation of colonoscopy 2–5 years prior to the earliest CRC diagnosis in the family if it occurs before the age of 30 years.

Collectively, these studies support a more intensive surveillance for *MLH1/MSH2* carriers. Although, the optimal surveillance interval remains a matter of debate, recent insights from Aronson et al. and Sleiman et al. suggest a preference for intervals within the 1–2-year range for *MLH1/MSH2* carriers. In contrast, for *MSH6/PMS2* carriers, these studies, based on metachronous CRC investigations, provide additional rationale for a delayed initiation of surveillance with longer intervals.

### Cost-effectiveness studies

Two recent studies have explored the cost-effectiveness of colonoscopy surveillance in LS with the aim of determining the optimal strategy. Kastrinos et al. employed a Markov simulation model to analyze the impact of initiation time and intervals for each specific gene on CRC incidence and mortality, quality-adjusted life-years (QALY), and cost. According to their analysis, the optimal surveillance strategy was annual surveillance starting at the age of 25 years for *MLH1*, biennial surveillance starting at the age of 25 years for *MSH2*, surveillance every 3 years starting at the age of 35 years for *MSH6*, and surveillance every 5 years starting from age 40 years for *PMS2* carriers [55]. Later, Kang et al. used an optimization algorithm and a microsimulation model analyzing the entire LS spectrum. Overall, surveillance every 3 years was less effective at averting CRC deaths but required almost 40% fewer colonoscopies compared to more frequent intervals, making it the most cost-effective strategy. While the initiation of the surveillance was more cost-effective starting at 25 years of age for *MLH1* and *MSH2*, 30 years for *MSH6* and 35 years for *PMS2* carriers [56]. While both studies investigated the cost-effectiveness, their different results can be explained by the distinct methodologies and setting. The first study was conducted in the United States with a higher willingness-to-pay (WTP) threshold, using 100,000 U.S. dollars/QALY, while the second, conducted in Australia, set their WTP threshold to 30,000–50,000 Australian dollars/life-year saved. Another significant difference lies in the gene-specific methodology employed by Kastrinos and colleagues. Accordingly, these studies add more evidence towards a more precise approach; however, the differences in costs and WTP among different countries make it challenging to generalize these results.

### Metachronous colorectal cancer risk after surgery

Eikenboom et al. explored gene-specific metachronous CRC risk in 527 LS carriers from the Dutch Foundation for the Detection of Hereditary Tumors (StOET) database. Metachronous CRC rates were 27% for *MLH1*, 26% for *MSH2*, 14% for *MSH6*, and 16% for *PMS2* carriers. In this study, the authors aimed at assessing the gene-specific risk of metachronous CRC after partial colectomy and extensive colectomy (subtotal or total colectomy). As expected, partial colectomy was associated with a higher risk of metachronous CRC than extensive colectomy in *MLH1/MSH2* carriers (hazard ratio 1.97, 95% CI 1.04–3.73; *p* = 0.039). Interestingly, the risk of metachronous CRC did not differ between carriers of *MSH6/PMS2* variants who had partial colectomy and those of *MLH1/MSH2* variants who had extensive colectomy (1.14, 0.55–2.36; *p* = 0.72) [57]. This study supports partial colectomy followed by regular endoscopic surveillance for *MSH6/PMS2* carriers, aligning with current recommendations. Notably, for *MLH1/MSH2* carriers, while partial colectomy initially showed a higher risk of metachronous CRC, this difference was not observed when analyzing cases diagnosed from 2000 onward. This observation suggests that even in *MLH1/MSH2* carriers, partial colectomy followed by surveillance colonoscopy could be a valid option. Another point to highlight is that while the incidence of metachronous CRC may be higher for *MLH1/MSH2* carriers under partial colectomy, the overall survival was not different between partial and extensive colectomy. Similar observations have been reported in two other retrospective studies, where the populations were predominantly *MLH1/MSH2/EPCAM* carriers (> 95%), and no survival benefit was observed for extensive colectomy over partial colectomy [58, 59].

## Conclusions

Despite recent advancements, the definitive role of colonoscopy in LS has yet to be established. Current evidence of the variable effect of colonoscopy effectiveness depending on quality indicators in LS suggests that the full potential of colonoscopy has not been achieved. The advent of new technologies such as digital chromoendoscopy or AI can be transformative in the coming years. However, this aspect needs to be analyzed preferably prospectively in a collective effort to establish the potential preventive effect of colonoscopy and establish specific quality indicators. On the other hand, there is increasing evidence of gene differences in LS, and the importance of personalizing surveillance and treatment recommendations based on the affected gene. In this sense, it will be crucial to identify robust risk factors that determine the risk of cancer to identify the high-risk population that benefits most of preventive measures. The coming years are going to be very exciting with the results of the CAPP-3 study [60] that will establish the role of different doses of ASA as cancer prevention, as well as the results of the first trials evaluating the effectiveness and safety of preventive vaccines in LS [61–63].

## Data Availability

No datasets were generated or analysed during the current study.
